# The Use of Pestles and Mortars

**Published:** 1876-05

**Authors:** Thos. Fletcher


### ARTICLE XI.

### *The Use of Pestles and Mortars.*

The use of a pestle in a very small mortar for making amalgams and oxychlorides is undoubtedly a mistake. If a pestle is used it necessitates the use of a much larger proportion of fluid than is really necessary to make a stiff paste; but if instead of a pestle a small spatula is used, working materials to be mixed against the side of the mortar instead of the bottom, the action and the result are precisely the same as when the mixing is performed in the hand.

As the results obtained with almost every amalgam are so very much better when a very small proportion of mercury is used, this hint will be, no doubt, a valuable one to those who dislike mixing in the hand. Until I discovered this mode of working I was under the impression that very dry amalgams could not be properly mixed in a mortar without very great difficulty, but the use of a spatula instead of a pestle renders this, if anything, an easier matter in the mortar than in the hand. Small flat slabs are commonly used for mixing oxychlorides, but they are much easier to work and keep in a compact mass if a mortar and spatula are used, as the liquid cannot run away, and the powder cannot fly about, as it is liable to do on a flat plate.—*Thos. Fletcher.*

---

